# Overview of Foodborne Disease Outbreaks in Brazil from 2000 to 2018

**DOI:** 10.3390/foods8100434

**Published:** 2019-09-23

**Authors:** Jéssica A. F. F. Finger, Wilma S. G. V. Baroni, Daniele F. Maffei, Deborah H. M. Bastos, Uelinton M. Pinto

**Affiliations:** 1 Food Research Center (FoRC-CEPID), Sao Paulo 05508-080, Brazil; 2 Department of Food and Experimental Nutrition, Faculty of Pharmaceutical Sciences, University of Sao Paulo, Sao Paulo 05508-080, Brazil; 3 Department of Nutrition in Public Health, Faculty of Public Health, University of Sao Paulo, Sao Paulo 01246-904, Brazil; 4 Ceara State University, Fortaleza 60741-000, Brazil; 5 Department of Agri-food Industry, Food and Nutrition, Luiz de Queiroz College of Agriculture, University of Sao Paulo, Piracicaba 13418-900, Brazil

**Keywords:** epidemiological survey, foodborne illnesses, food contamination, food safety, public health

## Abstract

This study aimed to assess the foodborne diseases (FBD) outbreaks reported in Brazil between 2000 and 2018, based on data from the Brazilian Ministry of Health (official data) and from scientific literature. According to official data, 13,163 FBD outbreaks were reported in the country during this period, involving 247,570 cases and 195 deaths. The largest prevalence of FBD outbreaks was observed in the Southeast region of Brazil (45.6%). In most outbreaks it was not possible to determine the food implicated (45.9%) but among those identified, water was the most frequently associated (12.0%). The etiological agent was not identified in most outbreaks (38.0%), while *Salmonella* (14.4%) was the most frequently reported among those identified. Homes were the main site of FBD occurrence (12.5%). Regarding data obtained from the scientific literature, 57 articles dealing with FBD in the country throughout the same period were selected and analyzed. Based on these articles, mixed foods were the most prevalent in the outbreaks (31.6%), *Salmonella* spp. was the pathogen most frequently reported (22.8%) and homes were also the main site of FBD occurrence (45.6%). Despite under-notification, the records of FBD outbreaks that have occurred in Brazil in the past recent years show alarming data, requiring attention from health authorities. The notification of outbreaks is essential to facilitate public health actions.

## 1. Introduction

Foodborne diseases (FBDs) are considered an important and growing public health issue and represent a significant cause of morbidity and mortality worldwide. They are the result of ingestion of contaminated foods or beverages, mainly by a variety of bacteria or their toxins, viruses, and parasites [1].

Common FBD symptoms include nausea, vomiting, abdominal pain, diarrhea, lack of appetite, and fever. The intensity of these symptoms depends on many factors, such as the pathogen involved, infectious dose, health conditions of the affected individual, among others [2]. The fact that many types of FBD trigger similar symptoms hinders the correct diagnosis. In addition to public health problems, FBD can cause significant economic losses since they may result in incapacity for work, costs with treatments, hospitalizations, and epidemiological investigations, as well as damages involving tourism and food sales [3,4].

The United States Centers for Disease Control and Prevention estimate that FBDs affect 48 million people annually, with 128,000 hospitalizations and 3000 deaths in that country [1]. Nevertheless, national and international reports consider that only a fraction of cases are documented, reported to public health authorities and recorded in official FBD statistics [1,5].

In Brazil, the occurrence of FBD outbreaks started to be reported to health authorities in 2000 through the National Epidemiological Surveillance System for Foodborne Diseases (Sistema Nacional de Vigilância Epidemiológica das Doenças Transmitidas por Alimentos—VE-DTA), under the responsibility of the Health Surveillance Office of the Brazilian Ministry of Health. Epidemiological Surveillance Agencies in each region/city of the country are responsible for the investigation of any FBD outbreaks and inclusion of relevant information into the VE-DTA, including the morbidity, mortality, and lethality, modes of transmission and contamination, incubation period, susceptibility, and resistance of individuals [2,6].

Although there are several surveillance systems for FBDs at the municipal, state and federal levels in many countries, it is estimated that only a fraction of the FDB outbreaks are reported to the appropriate authorities, due to the fact that a small proportion of affected individuals seek medical care [6,7]. Consequently, the lack of data hinders the assessment of the real dimension of the problem and the development of control strategies [8].

Despite the lack of data on the occurrence of FBDs, many studies point to an increase in the number of cases worldwide. Several factors may result in a higher number of cases, such as population growth, increased population of susceptible individuals, disorderly urbanization processes, and the need for large-scale production of foods [9,10,11]. According to the World Health Organization, most cases of FBDs could be avoided if preventive measures were taken in place throughout the food production chain, requiring effort by governments, the food industry, and consumers [5,12].

The present study mapped the FBD outbreaks that occurred in Brazil between 2000 and 2018, based on data reported by the Brazilian Ministry of Health and from scientific literature. These data are expected to contribute to the knowledge of FBD outbreaks occurring in the country, as well as to support food safety planning, promotion, prevention and control strategies.

## 2. Materials and Methods 

This descriptive epidemiological study consists of the search, classification, and analysis of data from the Brazilian Ministry of Health (official data) and from scientific articles dealing with FBD outbreaks in the country from January 2000 to December 2018. 

Official data were obtained from the Ministry of Health’s website and from the Electronic System of the Citizen Information Service (e-SIC). This is a system whereby any person or company may request access to information published by agencies at the Federal Executive bodies, including the Ministry of Health. The official data were organized into spreadsheets and classified according to the number of outbreaks/cases, exposed and ill individuals, deaths, distribution of FBD outbreaks by region, confirmatory criteria, food implicated, etiological agents, and site of occurrence.

The search for Brazilian scientific articles on FBD outbreaks was carried out on the following databases: LILACS (Literatura Latino-Americana e do Caribe em Ciências da Saúde), SciELO (Scientific Electronic Library Online), Scopus, Web of Science, PubMed, and Embase. The articles classified and selected in this study were those derived from research carried out in Brazil, available for consultation between June and July 2019. This analysis was performed by searching the databases using the following keywords in Portuguese: “doenças transmitidas por alimentos”, “surtos de doenças”, “investigação de surtos de doenças”, “Brasil”, and in English: foodborne disease, disease outbreak and Brazil. The selection was performed by carefully analyzing the titles, abstracts, keywords, and finally by reading the full text in order to define whether or not a publication meets the criterium of being a FBD outbreak described in Brazil, between the years 2000 and 2018. Data of selected publication were organized into spreadsheets and classified according to foods implicated in the outbreak, etiological agents, and site of occurrence (Supplementary Materials Tables S1 and S2).

Ethics approval and consent to participate: All procedures performed in the studies did not involve human participants. The data used for our study were openly available in the Brazilian Ministry of Health’s website and from the Electronic System of the Citizen Information Service.

## 3. Results

### 3.1. Data from the Brazilian Ministry of Health

**Figure 1:**
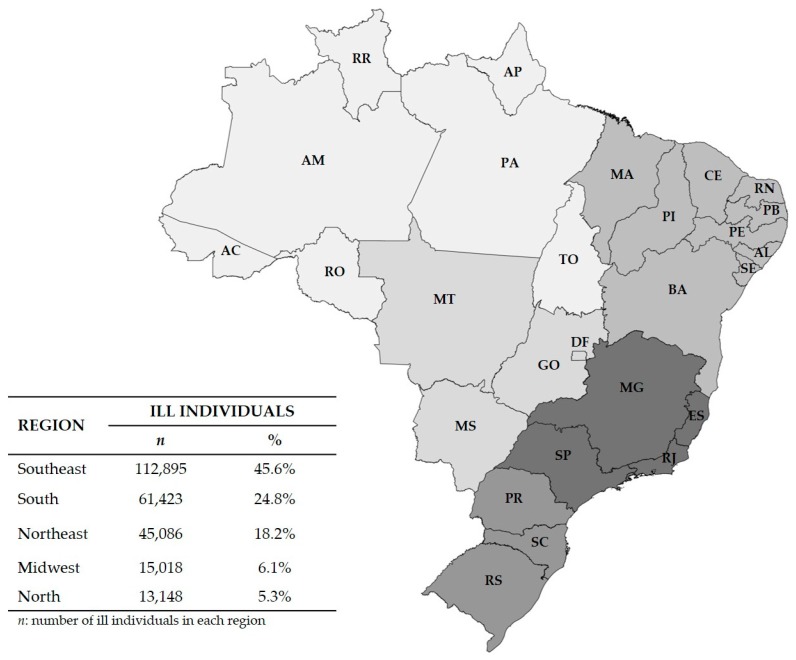
Distribution of ill individuals due to FBD outbreaks by region in Brazil, 2000 to 2018. The map was created by using MapChart. Southeast Region—ES: Espírito Santo, MG: Minas Gerais, RJ: Rio de Janeiro, SP: São Paulo; South Region—PR: Paraná, SC: Santa Catarina, RS: Rio Grande do Sul; Northeast Region—AL: Alagoas, BA: Bahia, CE: Ceará, MA: Maranhão, PB: Paraíba, PE: Pernambuco, PI: Piauí, RN: Rio Grande do Norte, SE: Sergipe; Midwest Region—DF: Distrito Federal, GO: Goiás, MT: Mato Grosso, MS: Mato Grosso do Sul; North Region—AC: Acre, AM: Amazonas, AP: Amapá, PA: Pará, RO: Rondônia, RR: Roraima, TO: Tocantins.

According to data from the Brazilian Ministry of Health, between 2000 and 2018, a total of 13,163 FBD outbreaks were reported to the Department of Health Surveillance, which estimates that 2,429,220 individuals had been exposed, resulting in 247,570 ill individuals and 195 deaths (Table 1). The highest incidence was recorded in the Southeast and South regions of the country, accounting for 70.4% of the reported cases. The Northeast region accounted for 18.2% of the cases, followed by the Midwest (6.1%), and the North regions (5.3%) (Figure 1). Most of these FBD outbreaks were confirmed after investigation based on epidemiological surveys (22.7%), clinical analyses (13.2%), bromatological analyses (10.1%), and epidemiological–clinical–bromatological analyses (8.8%) (Table 2).

Of the 13,163 outbreaks reported, it was not possible to determine the food implicated in most of them (45.9%) (Table 2). Among those identified, water was the most frequently associated vehicle within these outbreaks (12.0%), followed by mixed foods (10.4%), multiple foods (9.8%), and eggs (6.9%). When evaluated according to region of occurrence, the Northeast, Southeast, and Midwest regions showed water as the main source of FBD outbreaks. Multiple foods were the most frequently implicated in the North region, and eggs and egg products in the South region. 

Regarding etiological agents, the pathogen was not identified for most outbreaks (38.0%) (Table 2). Among those identified, *Salmonella* spp. (14.4%), Rotavirus (9.9%), and *Escherichia coli* (7.4%) were the most frequently reported. Other microorganisms were also mentioned, although in a lower proportion, such as *Staphylococcus aureus* (6.4%), *Bacillus cereus* (3.3%), and *Clostridium perfringes* (2.3%). Homes were pointed out as main site of occurrence in most outbreaks (12.5%), followed by daycare/school (10.6%), and restaurants/bakeries (9.3%) (Table 2).

### 3.2. Data from the Scientific Literature

The analysis of the databases resulted in the selection of 57 articles that met the purpose of this study (Supplementary Material 1). Regarding the main research topic, 30 (52.6%) articles dealt with a specific outbreak that occurred at a particular time and location, 18 (31.6%) carried out a study on FBD according to a specific etiological agent and 9 (15.8%) carried out a study on FBD in a specific region during a certain time. Only one study on the overall burden of FBD outbreaks occurring in the country was found, although addressing a shorter period (2007–2017). Additional data obtained from each scientific article, including number of cases and deaths when available, are shown in the Supplementary Material Table S2.

Mixed foods were the most frequently types associated with these reported outbreaks (31.6%), followed by water (21.1%) (Table 3). Regarding etiology, most of these studies focused on FBD outbreaks caused by *Salmonella* spp. (22.8%), followed by *Trypanosoma cruzi* (14.0%), and Norovirus (12.3%). However, in 5.3% of these studies the etiological agent was not identified (Table 3).

Most of these studies pointed out homes as the main site of FBD occurrence (45.6%), followed by restaurants (7.0%), workplaces (7.0%), events (3.5%), hospitals (1.8%), asylums (1.8%), and ships (1.8%). In 31.6% of these studies the site of occurrence was not identified (Table 3).

## 4. Discussion

Foodborne diseases represent one of the most common and important public health issues worldwide. According to the World Health Organization, 23 million people in the Europe Union (EU) become ill and 5000 die every year due to FBDs [15]. The Centers for Disease Control and Prevention estimates that FBDs affect 48 million people annually, with 128,000 hospitalizations and 3000 deaths in the United States of America (USA) [1]. 

In Brazil, little is known about the epidemiological profile of FBDs, since only a small number of cases are notified to food inspection and health agencies. The number of individuals that became ill (*n* = 247,570) and died (*n* = 195) due to FBDs reported in the country during the period covered in this study (2000–2018) is dramatically lower than that annually estimated for the EU and the USA. The same behavior is observed when the average number of FBD outbreaks and ill individuals annually reported in Brazil (693 and 13,030, respectively) is compared to other countries. 

In Canada, it is estimated that every year about 4 million individuals are affected by foodborne illnesses, resulting in approximately 11,600 hospitalizations and 238 deaths [16]. In Australia, annual reports on FBDs in the country have been produced and published in Communicable Diseases Intelligence since 2001. In 2012, OzFoodNet (Foodborne disease surveillance and response across Australia) sites reported 2180 outbreaks of gastrointestinal illness affecting 40,547 individuals, resulting in 955 hospitalizations and 131 associated deaths [17]. In South China, from 2010 to 2016, a total of 138 FBD outbreaks were reported (mean of 19.7 outbreaks annually), involving 3348 cases and 46 deaths [18].

Delayed notification, lack of clinical and/or food sample collection, inadequate laboratory tests, and even a difficulty in contacting involved individuals generate gaps in obtaining more detailed and reliable data on the FBD outbreaks [19]. Consequently, the absence of the real dimension on the occurrence of these FBDs limit the understanding of their importance for public health [9,20].

The present study showed that the states located in the Southeast and South regions of Brazil have a higher proportion of reported outbreaks when compared to the states located in other regions. This is directly related to the number of cities and towns that have the Foodborne Diseases Epidemiological Surveillance System (VE-DTA) well implemented. In addition, most of the Brazilian population (42.1%) lives in the Southeast region of the country [13,14,21]. 

Most of the studies and reports on the FBD outbreaks registered in Brazil pointed to water, multiple/mixed foods, and eggs/egg products as the main sources of foodborne pathogens. Mixed foods are characterized as multi-ingredient preparations, which are more susceptible to contamination due intense manipulation and, consequently, can carry a higher risk of food poisoning, especially when they’re not prepared, stored, or cooked properly [9]. Water has also a significant role on the occurrence of FBD outbreaks and its contamination is directly related to the precariousness of water treatment. In Brazil, drinking water must comply with the Ministry of Health guidelines, which sets the absence of total coliforms and *Escherichia coli* per 100 mL of water [22]. However, 16.7% of the population in the country (about 35 million people) do not have access to treated water [23]. 

Bacteria were the most common cause of the FBD outbreaks reported in the country, *Salmonella* being the most frequently involved pathogen. Contamination of foods by this bacterium may occur along the production chain. Failures during food handling, including poor personal and environmental hygiene, storage at inappropriate temperatures, and cross contamination may increase the risk of contamination [24,25]. The main foods involved in the FBD outbreaks caused by this bacterium are raw eggs, egg products, meat products, and vegetables [26].

A study conducted by Callejón et al. [27] concluded that *Salmonella* was the leading cause (22.7%) of FBD outbreaks that occurred in several states of the USA during 2004 and 2012. Kozak et al. [28] studied FBD outbreaks in Canada from 2001 to 2009 and found that *Salmonella* was the main pathogen involved (50%). In the Barbados-Caribbean Region, from 1998 to 2009, bacteria were responsible for 91.7% of outbreaks and 87.4% of cases. The most common bacterial causes of the 24 outbreaks were *Salmonella* Enteritidis (70.8%), *S. aureus* (8.3%) and mixed infections (8.3%) [29]. On the other hand, in the United States, from 2000 to 2008, most illnesses were caused by norovirus (58%), followed by nontyphoidal *Salmonella* spp. (11%), *Clostridium perfringens* (10%), and *Campylobacter* spp. (9%) [30].

Homes were the main site of FBD occurrence, followed by restaurants and bakeries. According to the European Food Safety Authority, 95% of cases of FBD came from small outbreaks originating in households [31]. A study conducted by Ting-ting [32] in China showed that, between 2002 and 2011, most deaths due to FBD outbreaks occurred in homes. In another study conducted in China, Li et al. [18] showed that schools (42.7%) and homes (32.6%) were the main sites of FBD outbreaks. These findings highlight the importance of investment in sanitary conditions and education for the population. Day care centers and schools represented the second largest site of FBD occurrence. These places usually have concentrations of high-risk groups, i.e., young children [33]. Restaurants and bakeries also present an important role in FBD occurrence.

Only one out of the 57 articles selected and analyzed in this study addressed the overall burden of FBD outbreaks reported in Brazil: A review conducted by Draeger et al. [9]. Although their study covered a shorter period (2007–2017) than the present work, there were similarities between the results: The largest prevalence of FBD outbreaks was observed in the Southeast region of the country (41.3%); in most cases it was not possible to determine the implicated food (57.4%), but among those identified, mixed foods and water were the most prevalent (9.1% and 6.6%, respectively); etiology was not identified in most cases (38.0%) but among those identified *Salmonella* spp. was the most frequent (22.1%), and homes were the main site of FBD occurrence (38.3%).

Overall, FBD mapping provides subsidies for the development of political, educational, and legislative measures. It is a challenge for FBD surveillance teams to create measures that standardize reporting across all Brazilian regions, reducing differences between surveillance systems among different counties, and minimizing the time between reporting the outbreak and starting investigations. However, it is crucial that epidemiological reports become more frequent and reliable for appropriate preventive and monitoring actions to be able to avoid the occurrence of new outbreaks.

## 5. Conclusions

Based on data obtained in this study, the records of FBD outbreaks reported in Brazil underrepresent the reality of the problem in the country. However, they still show alarming data, which require attention from the health authorities. Although the number of cases reported in the country is lower than that reported in other parts of the world, such as the USA and the European Union, it is known that this difference may be due to underreporting. Hence, efforts to improve the Brazilian surveillance systems are necessary, as the notification of outbreaks is essential to facilitate public health actions.

## Figures and Tables

**Table 1:** Data of foodborne disease outbreaks reported in Brazil between 2000 and 2018.

Year	Outbreaks	Exposed Individuals	Sick Individuals	Dead Individuals
2000	545	31,943	9613	4
2001	897	211,228	15,706	5
2002	823	116,962	12,402	5
2003	620	688,742	17,981	4
2004	645	368,158	21,781	21
2005	923	241,991	17,279	12
2006	577	49,044	10,356	8
2007	683	25,195	11,635	11
2008	641	23,275	8736	26
2009	594	24,014	9407	12
2010	498	23,954	8628	11
2011	795	52,640	17,884	4
2012	863	42,138	14,670	10
2013	861	64,340	17,455	8
2014	886	124,359	15,700	9
2015	673	35,826	10,676	17
2016	538	200,896	9935	7
2017	598	47,218	9320	12
2018	503	57,297	8406	9
**Total**	**13,163**	**2,429,220**	**247,570**	**195**

Source: Brazil, 2016 [13] and Brazil, 2019 [14].

**Table 2:** Confirmatory criteria, foods implicated, etiological agents, and sites of foodborne disease occurrence in Brazil between 2000 and 2018.

Component	Individuals	
	*n*	%
**Confirmatory criteria**		
Inconclusive	111,914	45.2
Epidemiological survey	56,203	22.7
Clinical analyses	32,693	13.2
Bromatological analyses	24,969	10.1
Epidemiological-clinical-bromatological analyses	21,791	8.8
**Foods implicated**		
Not identified	113,571	45.9
Water	29,690	12.0
Mixed foods	25,834	10.4
Multiple foods	24,206	9.8
Eggs/egg products	17,075	6.9
Red meats	8772	3.5
Others *	28,422	11.5
**Etiological agents**		
Not identified	93,981	38.0
*Salmonella* spp.	35,743	14.4
Rotavirus	24,434	9.9
*Escherichia coli*	18,398	7.4
*Staphylococcus aureus*	15,724	6.4
*Bacillus cereus*	8213	3.3
Inconclusive	8135	3.3
Norovirus	6076	2.5
*Clostridium perfringes*	5761	2.3
*Shigella sonnei*	5035	2.0
Others **	26,070	10.5
**Sites of occurrence**		
Homes	30,964	12.5
Daycare/school	26,143	10.6
Restaurants/bakeries	22,965	9.3
Not identified	20,305	8.2
Events	18,898	7.6
Hospitals	7615	3.1
Asylums	1106	0.4
Scattered sites	119,574	48.3

Source: Brazil, 2016 [13] and Brazil, 2019 [14]. * Others: other types of implicated foods accounting for less than 2% each; ** Others: other etiological agents accounting for less than 2% each.

**Table 3:** Data from the scientific literature on foods implicated, etiological agents, and sites of foodborne disease outbreaks described in Brazil between 2000 and 2018.

Component	Studies	
	*n*	%
**Foods implicated**		
Mixed foods	18	31.6
Water	12	21.1
Uninformed	8	14.0
Red meats and poultry	6	10.5
Fish and seafood	4	7.0
Acai/acai juice	4	7.0
Eggs/egg products	2	3.5
Vegetables	2	3.5
Sugarcane juice	1	1.8
**Etiological agents**		
*Salmonella* spp.	13	22.8
*Trypanosoma cruzi*	8	14.0
Norovirus	7	12.3
Virus da Hepatite A	4	7.0
Fish Toxin	4	7.0
Rotavirus	3	5.3
*Clostridium botulinum*	3	5.3
Uninformed	3	5.3
*Bacillus cereus*	3	5.3
Others *	9	15.8
**Sites of occurrence**		
Residences	26	45.6
Uninformed	18	31.6
Restaurants	4	7.0
Workplaces	4	7.0
Events	2	3.5
Hospitals	1	1.8
Asylums	1	1.8
Ships	1	1.8

* Others: other etiological agents accounting for less than 4% each.

## Supplementary Materials

The following are available online at https://www.mdpi.com/2304-8158/8/10/434/s1, Table S1: Selected articles from the scientific literature; Table S2: Data obtained from the selected scientific articles.
